# Comparison of indicators of material circumstances in the context of an epidemiological study

**DOI:** 10.1186/1471-2288-11-108

**Published:** 2011-07-18

**Authors:** Thomas Matukala Nkosi, Marie-Elise Parent, Jack Siemiatycki, Javier Pintos, Marie-Claude Rousseau

**Affiliations:** 1 Epidemiology and Biostatistics Unit, Institut National de la Recherche Scientifique (INRS)-Institut Armand-Frappier, 531, boulevard des Prairies, Laval, Québec, H7V 1B7, Canada; 2 Department of Social and Preventive Medicine, Université de Montréal, Montréal, Québec, Canada; 3 Université de Montréal Hospital Research Centre (CRCHUM), Montréal, Québec, Canada

## Abstract

**Background:**

Since individual-level income is difficult to collect, investigators often rely on group-based measures derived from census data. No study has assessed the use of residential property values as an indicator of individual material circumstances. We aimed to compare two proxy indicators of material circumstances, one based on residential value and the other on median census tract income, to self-reported household income.

**Methods:**

We used data from a case-control study (1996-2002), restricting analyses to 676 residents of the Island of Montreal for whom the three indicators were available. The degree of discrepancy between the residential value index, census income, and self-reported household income - each in 5 categories - was estimated, along with overall and weighted Kappas.

**Results:**

When comparing residential value index and census income to self-reported household income, perfect concordance was observed for 38% and 30% of subjects, respectively; very good concordance, defined as ≤ 1 category difference, was observed for 76% and 69% of subjects, respectively. When compared to self-reported household income, overall and weighted Kappas showed stronger agreement with residential value index (weighted Kappa = 0.37, 95% CI: 0.32, 0.42) than with census income (weighted Kappa = 0.25, 95% CI: 0.20, 0.30).

**Conclusions:**

A residential value index may provide a measure of material circumstances that is closer to self-reported household income than the commonly used census income. Each indicator presents advantages and disadvantages, and their choice may depend on study objectives and feasibility.

## Background

Material circumstances, best measured by income and wealth [1], are central in health research [2], since they can be an important determinant of health and health services use [3]. Income is a measure of the financial resources available at one point in time, whereas wealth measures the accumulation of material resources [1,4]. Education and occupation are often used in health research [5], but these factors provide information on socio-economic dimensions different from material circumstances and thus cannot be considered as surrogate measures of the latter [6].

While in theory, the best way to obtain individuals' income information is to ask them directly; in practice people often do not wish to report it or the quality of response is doubtful. The problem can be more acute for some sub-populations, for instance according to gender, age, or ethnic origin. This makes it very difficult for health researchers to collect reliable individual-level income information for the populations studied [2]. A commonly used surrogate measure of individual income is the area-based mean or median household income. Such measures are available for census areas and have been used 'inter alia' in Canada and in the U.S.A [7-11]. The validity of area-based measures derived from national census data as surrogates of individual income has been investigated [2,10-13]. Census-based measures have been reported to provide valid information that can be used in health research without being invalidated by concerns regarding ecological fallacy [5,10]. However, some have observed a substantial discrepancy between area-based and individual-level income measures, and suggested that caution should be used in interpreting results from studies in which area-based measures are used as proxies for individual and household income [2,12,13].

Recently, authors from Montreal (QC, Canada) have suggested using area-based values of residential properties [14]. They derived average residential values for street blocks (areas smaller than census tracts) as an alternative approach to capture group-level information on material circumstances. In their study, Smargiassi et al [14] achieved a better control of confounding with area-based residential property values than with census tract-based income, suggesting that the former might capture material circumstances, which they refer to as socio-economic status, more accurately.

In order to further explore the potential usefulness of residential values in population-based research, we developed a residential value index, but at the individual rather than group level. Using data from a study conducted in Montreal, we aimed to compare this residential value index, as well as census-based median household income, to self-reported household income for classifying individuals' material circumstances.

## Methods

### Study population

We used data from a case-control study of environmental risk factors for lung cancer conducted in Greater Montreal in 1996-2002 [15]. This study included 738 men and 465 women with lung cancer diagnosed at all major Montreal-area hospitals, and residing in the Greater Montreal area, which includes the Island, as well as the North and South Shores. Population controls were randomly selected from electoral lists which are continually updated in Quebec, Canada. They are thought to represent nearly complete listings of Canadian citizens residing in the province. Controls were frequency-matched to the distributions of age, sex, and electoral districts (comprising about 40,000 electors) of lung cancer cases; there were 899 men and 614 women. Overall participation rate was 76%, yielding 2,716 subjects. For this specific analysis, we restricted the study sample to the 2,003 participants who were residents of the Island of Montreal. Ethical approval was obtained from each participating institution, and all subjects provided an informed consent.

### Interviews

Interviews were conducted by trained, bilingual (English/French) interviewers between 1996 and 2002. Over 76% of individuals responded for themselves, whereas surrogate respondents provided information for the other participants. Detailed information was collected on socio-demographic and lifestyle characteristics, including family income, residential history, ethnicity, country of origin, and lifetime smoking history, along with a wide range of potential environmental risk factors.

### Indicators of material circumstances

The three indicators of material circumstances were derived from different sources. The first one, "self-reported household income", was elicited at interview with the following question: "What was the approximate total income for all household members from all sources, before income taxes, in an average year during the last 5 years?" This information was available for 730 (36%) of the 2,003 study participants included in this analysis. The proportion of response to this question was much lower among men (15%) than among women (71%). For the purpose of our analyses, the original eight multiple choice answers were collapsed to five categories by combining some categories with very low numbers of subjects: < $20,000 (30% of sample); $20,000-$29,999 (24%); $30,000-$49,999 (21%); $50,000-$69,999 (13%), and; ≥ $70,000 (12%).

The second indicator, "residential value index", was derived from the 1995 residential property assessments of the City of Montreal (QC, Canada) that are used for municipal tax purposes. Residential values from 1995 were extracted using the participants' addresses at the time of interview. This older database was provided to us upon request, although the databases for the most recent property assessment rolls are publicly available online.

The property value reflects the market value on July 1 two years before the assessment role comes in effect. The market value is defined as the most probable selling price in a free and open market [16]. To determine the market value of a given property, the appraiser can use one of the three following methods, although the method used for a specific property is not recorded in the databases: 1) the comparison approach, using similar properties that have been sold; 2) the cost approach, which consists in adding a property's land value to the depreciated cost of the building (obtained by subtracting depreciation from the current replacement cost), and; 3) the income approach, based on capitalizing its net operating income at a rate stemming from similar properties sold [16]. The latter applies only to buildings with tenants or commercial property.

The municipal database of property value assessment includes all buildings and contains the monetary value attributed to each building and lot. It indicates the number of residential units in each building, and the proportion of the building's area identified as commercial, if any. To estimate the residential property value, the proportion of commercial space was subtracted from the total value of each building. For multiple residence buildings, at the exception of condominiums for which there was an individual evaluation per unit, there was no information available to attribute a specific value to different residences within one building. The residential value was then divided by the total number of residential units to estimate the average value of one unit. It was impossible to distinguish whether a residential unit was owned or rented, thereby preventing us to determine if the residence represented an expense or contributed to wealth. However, we assumed that there was a reasonably close relationship between the value of a given residential unit and the costs ensued to rent or own it and thus simply considered its value as a measure of what the participant was able to afford in terms of housing. For this study, we restricted our data collection of residential property values to residents of the Island of Montreal for whom these data were centralized in a database maintained by the City of Montreal, and easily accessible. Residential values were available for a total of 1,862 individuals, representing 93% of the 2,003 study participants living within the Island of Montreal. The continuous values of this index were divided into 5 categories following the observed distribution for self-reported income: respectively 30%, 24%, 21%, 13%, and 12% in categories 1 (lowest) to 5 (highest). The resulting cutoff points were: ≤ $42,102; $42,103-$66,629; $66,630-$94,975; $94,976-$133,176; ≥ $133,177.

Finally, the third indicator, "census income", was obtained from the 1996 Canadian census. A "census tract" is the elemental geographic unit used by Statistics Canada to report socio-demographic characteristics [17]. In 1996, there were 757 census tracts in the Montreal area (Census Metropolitan Area), each comprising an average of approximately 4,400 people [18]. Census tract data were extracted using the postal code for the subjects' residential address at the time of interview. The median household income for the census tract corresponding to each subject's residential postal code was used. This information was obtained for all 2,003 participants included in the current analyses. The resulting continuous values were categorized according to the observed distribution for self-reported income, as described previously. The resulting cutoff values for census income were: ≤ $25,781; $25,782-$31,434; $31,435-$39,255; $39,256-$49,776; ≥ $49,777.

### Statistical analysis

**Figure 1:**
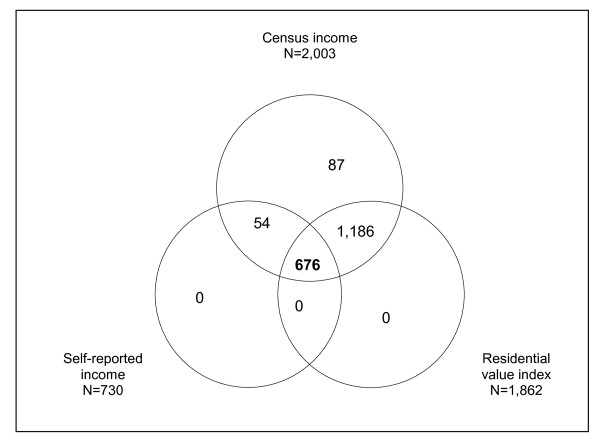
**Venn diagram depicting the availability of the three indicators of material circumstances among study subjects (N = 2,003)**.

Statistical analyses were restricted to 676 subjects residing on the Island of Montreal and for whom the three indicators of material circumstances were available, as depicted in Figure 1.

Firstly, the frequency distributions of the participants' selected characteristics and indicators of material circumstances were described. As mentioned previously, in order to compare the ranking of individuals according to the different indicators, the frequency distribution obtained for self-reported household income was used to categorize the residential value index and census income variables.

Secondly, the degree of discrepancy between self-reported household income and the other two indicators (residential value index, census income) was estimated. This was achieved by calculating the number of category differences (1 to 5) between each pair of indicators being compared.

Thirdly, pair wise correlations were estimated with the Spearman correlation coefficient, and agreement other than expected by chance was estimated with the overall and weighted Kappas and their 95% confidence intervals. Similarly to the Kappa coefficient (K), the weighted Kappa (K_w_) is adjusted for chance agreement given the marginal distributions [19]. Whereas K considers only complete agreement, K_w _was developed for use with categorical scales, in order to account for partial agreement by allowing disagreements of varying magnitude to be weighted accordingly [19-21]. In our case, linear weights were used for K_w_, thus attributing the same importance to a one-category difference, irrespective of whether it was between categories 1 and 2, or 4 and 5. Disagreements by a higher number of categories had a lower contribution to the K_w_, such that perfect agreement had a weight of 1, and differences of 1-4 categories had respective weights of 0.75, 0.50, 0.25, and 0 [22]. Various arbitrary guidelines exist for the interpretation of the strength of agreement. According to Landis and Koch, <0.00 represents "poor" agreement, 0.00-0.20 "slight" agreement, 0.21-0.40 "fair" agreement, 0.41-0.60 "moderate" agreement, 0.61-0.80 "substantial" agreement, 0.81-1.00 "almost perfect" agreement [23]. Fleiss suggested that Kappa values <0.40 should be regarded as indicating "poor" agreement, 0.40-0.75 "fair to good" agreement, and >0.75 excellent agreement [24]. All analyses combined cases and controls after verification, in stratified analyses, that the case or control status had little influence on the results.

## Results

Selected characteristics of participants are described in Table 1. The sample included a majority of women, who were more likely than men to have provided their income. Most subjects were of French ancestry, born in Canada, and self-respondents to the interview. One fifth of the men and one quarter of the women had post-secondary education. More women than men had never smoked. According to information from the property value assessment, our study population lived mainly in housing with two or more residential units (72%).

**Table 1:** Socio-demographic characteristics of study subjects

Characteristic	Males N = 177 (%)	Females N = 499 (%)	Total N = 676 (%)
Age (years)			
29-49	5.6	12.8	10.9
50-59	12.4	30.9	26.0
60-69	57.1	37.5	42.6
≥ 70	24.9	18.8	20.4
Marital status			
Married/cohabitating	75.1	52.3	58.3
Single	7.9	12.4	11.2
Separated/divorced	13.0	17.4	16.3
Widowed	4.0	17.8	14.2
Level of education			
Primary	38.4	27.5	30.3
High-school	41.8	47.1	45.7
Post-secondary	19.8	25.5	24.0
Ancestry			
French	69.5	70.9	70.6
Other	30.5	29.1	29.4
Respondent			
Self	88.1	86.6	87.0
Other	11.9	13.4	13.0
Country of origin			
Canada	78.5	80.4	79.9
Other	21.5	19.6	20.1
Smoking status			
Never	11.3	31.6	26.5
Ex-smoker	62.1	44.1	48.8
Current smoker	26.6	24.0	24.7

The self-reported household income variable was ordinal while the others were continuous. We demarcated the categorical boundaries for the residential value index and the census income index so that the marginal distributions of those variables would approximate that of self-reported household income (i.e., 30%; 24%; 21%; 13%; 12%). The resulting frequency distributions of the three indicators of material circumstances are presented in Table 2.

**Table 2:** Frequency distribution of indicators of material circumstances

Indicators	Males N = 177 (%)	Females N = 499 (%)	Total N = 676 (%)
Self-reported household income			
Category 1: < $20,000	20.3	33.7	30.2
Category 2: $20,000-$29,999	31.6	21.4	24.1
Category 3: $30,000-$49,999	26.6	19.0	21.0
Category 4: $50,000-$69,999	13.0	12.4	12.6
Category 5: ≥ $70,000	8.5	13.4	12.1
			
Residential value index			
Category 1: ≤ $42,102	28.2	30.7	30.0
Category 2: $42,103-$66,629	27.1	22.8	24.0
Category 3: $66,630-$94,975	22.6	20.4	21.0
Category 4: $94,976-$133,176	13.6	12.8	13.0
Category 5: ≥ $133,177	8.5	13.2	12.0
			
Census income			
Category 1: ≤ $25,781	29.9	30.7	30.5
Category 2: $25,782-$31,434	22.0	24.4	23.8
Category 3: $31,435-$39,255	24.9	19.2	20.7
Category 4: $39,256-$49,776	13.0	13.0	13.0
Category 5: ≥ $49,777	10.2	12.6	12.0

Table 3 presents the degree of discrepancy between five-category distributions of residential value index, census income and self-reported household income. Perfect concordance between residential value index and self-reported household income was observed for 38% of subjects, while the corresponding figure between census tract income and self-reported household income was 30%. Very good concordance, defined as no more than one category difference, was observed for 76% of subjects when comparing the residential value index to self-reported household income and 69% when comparing census tract to self-reported household income. In both of these comparisons, the discordant observations were equally distributed between under- and overestimation.

**Table 3:** Proportion and number of subjects according to the degree of discrepancy between residential value index, census income, and self-reported household income (N = 676)

Comparison	Number of category differences							
	
	Underestimation of Self-reported household income			Perfect concordance	Overestimation of Self-reported household income			Total
		
	≤-3	-2	-1	0	1	2	≥3	
Residential value index vs. Self-reported household income								
%	3.1	8.9	19.1	38.3	18.5	8.3	3.9	100
(n)	(21)	(60)	(129)	(259)	(125)	(56)	(26)	(676)
								
Census income vs. Self-reported household income								
%	5.3	9.6	20.6	29.6	19.1	10.9	4.8	100
(n)	(36)	(65)	(139)	(200)	(129)	(74)	(33)	(676)

Spearman correlation coefficients and Kappa values for the five-category variables are presented in Table 4. The correlation was stronger between residential value index and self-reported household income (r_Spearman _= 0.52) than between census and self-reported household incomes (r_Spearman _= 0.36). Overall and weighted Kappa values were relatively low (<0.40), although weighted Kappa values were slightly higher, as expected. When compared to self-reported household income, both the overall and weighted Kappa showed stronger agreement for residential value index (K_w _= 0.37, 95% CI: 0.32-0.42) than for census income (K_w _= 0.25, 95% CI: 0.20-0.30). The absence of overlap in the confidence intervals both for the overall and weighted Kappas indicated stronger agreement when using residential value index, e.g., credible values based on the 95% CI were 0.32-0.42 for K_w _for the agreement between residential value and self-reported income, whereas they varied from 0.20-0.30 for the agreement between census and self-reported incomes.

**Table 4:** Correlation and agreement between residential value index, census income, and self-reported household income (N = 676)

	Spearman correlation coefficient	Overall Kappa (95% CI)	Weighted Kappa (95% CI)
Residential value index vs. Self-reported household income	0.52	0.21 (0.16 - 0.25)	0.37 (0.32 - 0.42)
Census income vs. Self-reported household income	0.36	0.09 (0.05 - 0.14)	0.25 (0.20 - 0.30)

In our study, subjects were interviewed from 1996 to 2002. Census income was based on the 1996 Canadian census, whereas the residential value index was derived from the 1995 property assessment role from the City of Montreal. For subjects recruited towards the end of the study, the comparison could possibly have been improved by using the next census and property assessment role, both from 2001. To assess the impact of timing of data used for the census and residential value indicators, we carried out a sensitivity analysis excluding 256 participants interviewed after 1998 (N = 420). We also tested the impact of having included subjects for whom a proxy respondent provided the information. In both sensitivity analyses, the K_w _remained very similar suggesting that neither the use of 1995 property assessment role and 1996 census nor the use of proxy respondents had a strong influence on the results.

## Discussion

In our study, there was a very good concordance (defined as no more than one category difference) for a majority of the participants between self-reported household income, and both the residential value index (76%) and census income (69%). Perfect concordance with self-reported household income was higher for residential value index (38%) than for census income (30%). In addition, although Kappa values were generally low, weighted Kappa values were relatively higher between residential value index and self-reported household income than between census and self-reported household incomes (0.37 versus 0.25). This finding suggests that the residential value index may provide a slightly more accurate proxy for self-reported household income than census income, although the strength of agreement was in the "poor to fair" range. Our results also showed that for both residential value index and census income, values discrepant by more than one category were equally distributed between under- and overestimation of self-reported household income.

Overall, our study findings are in line with results from other similar studies suggesting relatively good concordance between self-reported household income and census tract income [5,10]. The census-based methodology was shown to be a valid and useful approach to overcoming the absence of socioeconomic data in most US medical records [11].

Nevertheless, when considering only the level of agreement between census and self-reported household incomes, we have observed relatively low levels of both overall and weighted Kappas. These findings are consistent with studies in which potential misclassification of income was investigated by comparing individual versus area-level measures of socioeconomic status, and that have suggested poor agreement between self-reported household income and census tract income [2,12,13,25]. It was suggested that the individual and census-based income might measure different constructs [12], and that aggregate measures should not be interpreted as individual ones [26].

An important finding from our study is that the residential value index was found to have slightly better agreement with self-reported household income than did census income. It might be partly due to the individual nature of both the residential value index and the self-reported income, as opposed to the area-level of the census income measurement. To our knowledge, our study is the first to use residential values at the individual level as an indicator of material circumstances. However, our results are in agreement with the observations from the study by Smargiassi and colleagues who used group-level residential values for a geographical area smaller than census tract, though not at the individual level [14].

### Limitations and strengths

Limitations and strengths of this study are mainly related to the three indicators of material circumstances. We elected to use the self-reported household income as the base of comparison. However, we are cognizant that it is not necessarily a 'gold standard'. The self-reported household income was obtained from the participants' answers, and as such could be inaccurate or even biased. Many individuals are reluctant to disclose their income. Small differentials between true and self-reported household income would be expected to have little influence on results. Indeed, even if the participants slightly over- or underestimated their income, they would possibly remain within the same income category or be allocated in an adjacent one. However, any large discrepancy between true and self-reported household income could have introduced serious misclassification. In addition, selective response to the household income question could have introduced bias if agreement varied according to the factors that affected response. To verify this, we assessed whether census income and residential value differed according to sex and self-report (or not) of household income. When stratifying the entire study sample by sex and presence/absence of self-reported household income, the median household census income was $33,313 and $33,312 respectively among men and women who responded, $30,300 among non-respondent men, and $30,950 among non-respondent women, suggesting a slightly but not markedly lower income among those who did not respond to the household income question, and little difference according to sex. There were no differences in distributions of residential value by sex and by presence/absence of self-reported household income (data not shown). This suggests that the relatively low response rate to the household income question and the larger proportion of women who responded is not likely to have strongly influenced our results.

Census tracts are demarcated by Statistics Canada with a view to creating socially homogeneous units. Still, there is some financial heterogeneity within census tracts; even neighbors may have different financial means. Thus median census tract income is certainly an imperfect indicator of the financial means of each individual in the census tract.

For municipalities in which property assessment rolls exist and can be publicly accessed, there are a number of advantages from using the residential value information. The residential municipal evaluation is intrinsically adjusted in terms of the degree of investment in maintaining the property, so a higher value will reflect, to a certain extent, the availability of funds for maintenance. The residential value index for participants is expressed as the average value of one residential unit. It has the advantage of taking into account the disparities in the quality of neighborhood and the housing conditions that prevail [27]. Also considered are the specific characteristics of the neighborhood and several factors such as the size of the house, type and materials used in its construction, physical environment, proximity to public transportation, shops and services, noise impacts, access to major roads and hospitals, which contribute to determine the value of a property. The residential value index is thus an indicator of great interest, because it represents the residential choice which is determined, in part, by the economic power of the household [27].

A few issues deserve attention with respect to the residential value index. Firstly, we could not assign a value to persons residing in retirement and other community homes. However, since we were able to document the residential value for 93% of those living on the Island of Montreal, these represent a small fraction of the entire population. Secondly, to our knowledge, the specific method used by the appraiser to establish the value of a given property is not documented in the publicly available databases. It is unclear whether the choice of appraisal method had an impact on the property values per se. Thirdly, for this study we restricted our sample to residents of the Island of Montreal for whom residential values were readily available as a result of a recent administrative restructuring and city merger. This information is available in other municipalities, and thus could eventually be gathered for the entire study. The Island of Montreal contains a high proportion of buildings with multiple units, many of them rentals, for which residential value is more difficult to assign. For this reason, we believe that our estimates of agreement are more conservative than if we had been able to also consider the suburbs surrounding the Island of Montreal, comprising a greater proportion of single unit dwellings. Fourthly, a limitation of the residential value index is that it was not possible to distinguish the surface area of each unit for buildings with more than one unit. Misclassification could have resulted, especially for residents of multiple dwelling buildings who were attributed the average value of a dwelling. Finally, no distinction could be made between owners and renters, possibly leading to an underestimation of financial availability for the former. Some of these potential limitations could be mitigated by collecting additional information at the time of interview concerning the owner or renter status of the subjects and the size of the dwelling when the property is part of multiple units.

The public availability of residential property values is a local administrative matter, but a non-exhaustive search on the internet allowed us to identify quite a few large cities, counties and countries where such data are accessible online [28-30] or where they exist and could possibly be accessed after an official request [31-35]. Since individual residential property values have the potential to approximate individual income at least as well as either census-based income or group-level residential property values, it appears to be a potentially valuable source of information that investigators should consider collecting, instead of or in addition to other indicators of material circumstances.

## Conclusions

Overall, our results suggest that using a residential value index may provide a measure of material circumstances that is closer to self-reported household income than the commonly used census income. Each indicator presents, however, advantages and disadvantages and their use may depend on study objectives and feasibility. In some situations a choice may be indicated, whereas in others the use of several complementary indicators will allow for a more comprehensive measurement of material circumstances, taking into account both income and wealth.

Finally, given the generally low Kappa values observed in our study, further research on the validity of a residential value index as an individual indicator of material circumstances and comparisons between individual and area-level residential values are warranted.

## Competing interests

The authors declare that they have no competing interests.

## Authors' contributions

All authors have contributed substantially to the manuscript. TMN was responsible for data analysis and interpretation, and for writing the first version of the manuscript. MEP and MCR were involved in the conception and design of the analytical strategy, interpretation of the data and critical revisions of the manuscript. JP provided the initial idea for the analysis, participated to the conception and design of the analytical strategy, and contributed critical revisions to the manuscript. JS conceived and realized the original study, was involved in data interpretation, and provided critical revisions to the manuscript. All authors have approved the final version of the manuscript.

## Pre-publication history

The pre-publication history for this paper can be accessed here:

http://www.biomedcentral.com/1471-2288/11/108/prepub
